# Rating the Raters: Legal Exposure of Trustmark Authorities in the Context of Consumer Health Informatics

**DOI:** 10.2196/jmir.2.3.e18

**Published:** 2000-09-19

**Authors:** Nicolas P Terry

**Keywords:** Internet, Medical Informatics Applications/legislation and jurisprudence, Rating, Consumer Health Informatics, Risk management, Liability, Legal

## Abstract

There are three areas of potential legal exposure for an organization such as a trustmark authority involved in e-health quality rating. First, an e-health provider may make a complaint about negative or impliedly negative ratings rendered by the ratings body (false negative). Typically, a negative ratings complaint would rely on defamation or product disparagement causes of action. In some cases such complaints could be defended on the basis of absence of malice (US). Second, the rating body might render a positive rating on e-health data that a third party allegedly relied upon and suffered injury (false positive). While the primary cause of action would be against the e-health data provider, questions may arise as to the possible liability of the trustmark authority. For example, some US liability exposure is possible based on cases involving the potential liability of product warrantors, trade associations, and certifiers or endorsers. Third, a ratings body may face public law liability for its own web misfeasance. Several risk management approaches are possible and would not necessarily be mutually exclusive. These approaches will require careful investigation to assess their risk reduction potential and, in some cases, the introduction of legislation.

## Introduction

The avowed, logical, and admirable purpose of a trustmark system such as MedCERTAIN is to "establish a fully functional self- and third-party rating system enabling patients and consumers to filter harmful health information and to positively identify and select high quality information" [1]. A ratings system inserts itself into the meta-information structure surrounding consumer choice. The motivation of a trustmark authority may be totally altruistic, specifically: to reduce informational asymmetry between patients and providers of health care, drugs, or advice. However, any such rating authority joins the ranks of infomediaries that increasingly will be exposed to legal liability for the occurrence of risks associated with e-health services.

It may seem that there is something intrinsically negative, even self-defeatist in injecting notions of legal liability at this stage in the development of health informatics trustmark authorities. Any such caustic view will no doubt be compounded when the source of such notions is US law, a system not renowned in the rest of the world for any exercise of self-restraint when it comes to the imposition of liability. As a famous US jurist once put it "[as] a litigant I should dread a lawsuit beyond almost anything else short of sickness and death" [2]. However, any partisan reaction should be resisted - the whole process of rating is premised on a desire for improved quality; and those who rate must be subject to the same high standards, while at the same time protected from overt or exaggerated disincentives to perform their evaluative tasks.

This paper primarily examines potential liability under US law. The choice of US law is deliberate and should not be dismissed as parochial, the product of regional bias or, worse, some clumsy attempt at legal colonialism. US tort law (delictual) liability for inaccurate information is more mature than that found in other countries (a fact that should not necessarily be equated with optimal results). Further, until the Internet and (and hence e-commerce and e-health business models) take on more of a Eurocentric focus (itself unlikely until the second half of the decade), the majority of e-health sites will be US (or US-centric), while the majority of those who rely on trustmarks likely will be US residents and an even larger number will look for legal relief before US courts [3].

## Liability Scenarios

**Figure 1:**
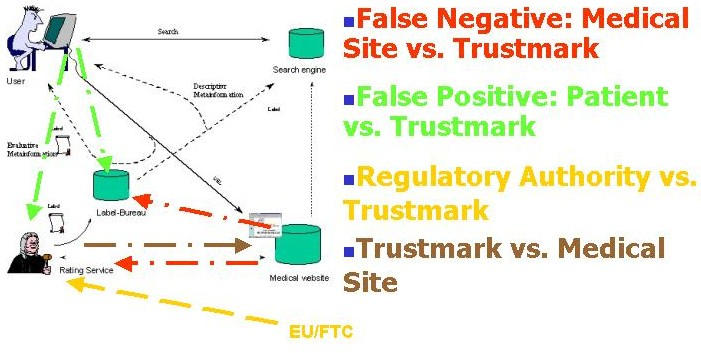
Four Trustmark Liability Scenarios (modified after [3])

A trustmark authority likely would perform several functions. First, it facilitates self-rating by, say, consumer health sites (providing criteria, link pages, or "tokens" to symbolize compliance). Second, it provides independent external evaluation of the content on sites, a process that can involve either "whitelisting" or "blacklisting." Third, and assuming a decentralized model, the central trustmark authority itself promotes inspection and trustmark qualifying activities much like a franchisor or intellectual property holder. Poor quality performance of these functions could negatively impact the reputation and economic health of the medical site evaluated (false negative cases) or the economic or physical well being of a consumer or patient (false positive cases). Finally, the authority itself generates and provides information about its own or rating systems generally, and may perform standard web functions such as user data collection or profiling and cookie generation (issues primarily of interest to public authorities) (see Figure 1).

### False Negative Ratings

Negative ratings (or "blacklisting") could dramatically affect the level of traffic to a medical advice web site (or its "stickiness" in the case of metadata-supplied rating information), and hence its ability to attract advertising or financing. Sites that complain of an allegedly incorrect low rating likely would argue that the trustmark authority is liable for damages on some type of product disparagement (probably the most famous US case of this type is *Bose Corp. v. Consumers Union*, 466 U.S. 485, 80 L. Ed. 2d 502, 104 S. Ct. 1949 (1984) in which a product manufacturer that complained about a review in a well-known consumer magazine was held to have to prove malice or reckless disregard of the truth) or defamation theory [4]. There are several, far more tenuous theories that may be raised by rated sites. These might include arguments as varied as:

- "trespassing" by the trustmark authority for entering or linking without consent
  (e.g., *Ebay, Inc. v. Bidder's Edge, Inc.*, 100 F. Supp. 2d 1058 (N.D. D. Cal. May 24, 2000) in which the auction web site was granted a preliminary injunction to halt software robot examination of its site on behalf of auction aggregating site)
- trademark infringement or dilution
  ( *New Kids on the Block v. New America Pub., Inc.*, 971 F.2d 302 (9th Cir. 1992) which ruled that fair use applied when newspaper used trademark to identify pop group and not to imply the group's endorsement. Court further noted that a competitor could use a rival's trademark in advertising for profit if the use was not false or misleading and did not implicate the source-identification function of the trademark. Also applied to web linking and searching in *Playboy Enterprises, Inc. v. Netscape Communications*, 2000 U.S. Dist. LEXIS 13418 (C.D. Cal. September 12, 2000))
- anti-competitive activity
  (an antitrust claim would be extremely difficult to sustain absent evidence that, for example, the trustmark authority became dominated by whitelisted sites and such created a barrier of entry to the e-health market)

Not surprisingly "truth" remains the best defense to the most likely type of action - that for defamation. However, it is not always the most cost-effective defensive approach. Under US law it is generally the case that "in a suit by a private plaintiff involving a matter of public concern... allegedly defamatory statements must be provably false, and the plaintiff must bear the burden of proving falsity, at least in cases where the statements were directed towards a public audience with an interest in that concern" [5] . Yet, burden of proof of falsity aside, US law offers some powerful defensive arguments. As is well known, the United States Supreme Court in *New York Times Co. v. Sullivan* [6], held that a public official could not recover for libel absent a showing of "actual malice" by the newspaper publisher. While that principle does not fit the trustmark authority scenario exactly, later cases have made clear that "[i]t is speech on matters of public concern that is at the heart of the First Amendment's protection" [7].

As a result, there seems general agreement that what is known as a "qualified privilege" will be extended to non-profit organizations such as trustmark authorities that undertake to rate services supplied by others [8]. This is particularly the case where it is *public* figures or organizations that are being rated (e.g., *National Foundation for Cancer Research v. Council of Better Business Bureaus*, 705 F.2d 98 (4th Cir. 1983), which held that a non-profit that engaged in mass solicitation efforts and declared a goal of making itself a "household name" was a "public figure" thereby erecting the Sullivan obstacles to defamation liability). In general terms such defensive categorizations would compel a plaintiff under US law to prove *actual malice*- that the trustmark authority gave an inaccurate rating based on knowledge of the true facts or reckless disregard of the accuracy of the rating (e.g., *Elite Funding Corp. v. Mid-Hudson Better Business Bureau*, 165 Misc.2d 497, 629 N.Y.S.2d 611 (N.Y. Sup. Ct. 1995), in which the brokerage claimed BBB's "unsatisfactory" rating was defamatory, held: (1) statement that brokerage had "unsatisfactory" record based on pattern of not responding to customer complaints was true and therefore not defamatory, and (2) even assuming challenged statements were not true, brokerage failed to produce evidence of express malice). This would not be an easy burden for the e-health site protagonist. However, narrow windows of vulnerability would open up if, for example, the trustmark authority lacked internal quality control procedures or had a record of inconsistent criteria or results (an issue that might well arise given a decentralized ratings system).

### False Positive Ratings

Whitelisting cases typically will involve actions brought by patients alleging injury because of reliance on data or treatment extracted from a medical advice web site previously rated by the trustmark authority. The authority becomes involved if the patient alleges that reliance on the trustmark influenced the choice of advice site and so, albeit indirectly, caused the injury complained of.

An initial analytical step is to examine the potential liability of web-based information and advice sites. In general terms these have little liability exposure under US law [9] . Decided cases suggest that courts are unwilling to impose duties on either authors or publishers. For example, in *Birmingham v. Fodor's Travel Publications, Inc.* [10], the court considered the potential liability of the publisher of a travel guide that failed to mention the dangerous ocean surf conditions at a beach resort. The publisher was held to be under no duty to warn a reader because "absent guaranteeing or authoring the contents of the publication, a publisher has no duty to investigate and warn its readers of the accuracy of the contents of its publications" [11]. (See also *Smith v. Linn*, 563 A.2d 123, 126 (1989), in which a reader died of complications arising from the liquid protein diet featured in a book; and *Walters v. Seventeen Magazine*, 241 Cal. Rptr. 101 (Ct. App. 1987) in which plaintiff contracted toxic shock syndrome allegedly as a result of using a tampon advertised in the defendant's magazine). In cases where the defendant is more closely tied to the origination of the flawed content, courts have been swayed by constitutional (freedom of speech) arguments. For example, in *Herceg v. Hustler* [12], the plaintiffs' 14-year old son took his own life attempting the practice of autoerotic asphyxia, having read about the practice in a magazine article. Citing well-known First Amendment case law, the court found the speech was protected. At first sight it would seem that many medical advice and treatment sites are commercial in nature and so-called "commercial speech" is given only limited protection by the First Amendment. However, even dangerous content will not qualify as "commercial" just because a web site accepts advertising or even is paid to serve the content [13].

At common law, therefore, it is clear that even US courts have circumscribed a relatively narrow window of private law liability for print "advice" content, and there are no indications that cyberspace content will attract any more stringent liability. Ironically, however, the trustmark authority could be under greater threat of legal liability than the underlying medical data sites that it rates. This is certainly the case from a purely practical perspective. High visibility, "brand-name" advice sites are more likely to put considerable resources into their own quality assurance programs. They are also likely to be highly protective of their brand and settle all but the most frivolous or speculative lawsuits. In contrast, the low-resource, high-risk site likely will "fold" at the first sign of litigation, leaving the trustmark authority as the most exposed potential defendant.

Beyond purely practical considerations, US torts doctrine suggests that the relative safety with which at least non-reckless advice sites operate might not extend to a trustmark authority. This distinction primarily is based on a recognition that the trustmark authority has voluntarily undertaken a role that it knows and intends the third-party consumer to rely upon. Of greatest potential concern is the cause of action summarized in §324A of the RESTATEMENT (SECOND) OF TORTS:

One who undertakes, gratuitously or for consideration, to render services to another which he should recognize as necessary for the protection of a third person or his things, is subject to liability to the third person for physical harm resulting from his failure to exercise reasonable care to protect his undertaking, if

(a) his failure to exercise reasonable care increases the risk of such harm, or

(b) he has undertaken to perform a duty owed by the other to the third person, or

(c) the harm is suffered because of reliance of the other or the third person upon the undertaking.

In cases involving certifiers or endorsers of defective products, this theory has been held sufficient to base an action against the certifier. For example, in *Hempstead v. General Fire Extinguisher Corporation* [14], Underwriter's Laboratories, a well-known non-profit testing laboratory, was held potentially liable after a whitelisted fire extinguisher exploded. The court noted: "The alleged failure of Underwriters to exercise reasonable care in approving the design of the extinguisher has obviously increased the risk of harm to plaintiff over that which would have existed if reasonable care had been exercised" (approved of by the court in *Arnstein v. Manufacturing Chemists Association, Inc.*, 414 F. Supp. 12 (E.D. Pa. 1976), also positing potential liability based on the closely related RESTATEMENT (SECOND) OF TORTS § 323).

In a case where the trustmark authority does "passive" rating by, for example, making its trustmark available to an e-health site for self-rating, then a liability argument likely would track the case of *Hanberry v. Hearst Corp.* [15], in which a shoe manufacturer utilized a magazine's "Good Housekeeping Consumers' Guaranty Seal" (cf. *Yanase v. Automobile Club of So. Cal.*, 212 Cal.App.3d 468, 260 Cal.Rptr. 513 (1989) which held that the family of an auto club member killed in motel parking lot was not owed a duty of care with respect to neighborhood safety or security measures at motels listed and rated in guide). More recent cases are consistent, continuing to enlarge the pool of potential defendants to include intellectual property licensors and trade associations that become involved in certifying or endorsing the activities or representations of others (e.g., *Torres v. Goodyear Tire*, 786 P.2d 939 (Ariz. 1990); *King v. National Spa*. 570 So.2d 612 (Ala. 1990)). Obviously, a proposal for a trustmark authority such as MedCERTAIN posits several different approaches for the delivery of evaluative metainformation [3]. At least as a working hypothesis it could be argued that the closer the trustmark authority integrates itself at a commercial or technological level with the underlying medical data suppliers, the greater will be its liability exposure.

It must be emphasized that even under US law a trustmark authority's exposure under such theories is somewhat limited. While the plaintiff may be able to point to a recognized cause of action, it does not follow that the trustmark authority ultimately would be held liable. Generally, data is not considered a "product" for the purposes of applying products liability doctrine (although if data were considered a "product," a trustmark authority might be exposed under Article 3, 1. of the products liability directive, where a "producer" means the manufacturer of a finished product, the producer of any raw material, or the manufacturer of a component part and any person who, by putting his name, trade mark, or other distinguishing feature on the product presents himself as its producer [17]). As a result, the plaintiff would still have to prove that the authority failed to exercise reasonable care and that such negligence caused plaintiff's injury. Specific acts of negligence that might be alleged by consumer plaintiffs could include failure by the authority to follow its own internal ratings criteria or, somewhat less convincingly, a trustmark authority's failure to enforce its intellectual property claims against sites fraudulently using the trustmark.

### Public Law Liability

Public law liability probably is the least of the concerns of a non-profit trustmark authority. Nevertheless, such an authority by its nature will generate and publish information about its own functions and practices. Particular care would be needed if the authority accepted any form of advertising or engaged in any for-profit activities. In particular, there could be public law exposure if rated sites could in any way "buy" disproportionate visibility, and such practices are not clearly disclosed to users (a practice that would also increase civil liability exposure).

It should also be assumed that a trustmark authority would perform standard web functions such as data collection and cookie generation. Such functions may be performed within its non-profit mandate, for example, to establish traffic patterns to justify its continued funding by public or other entities. Nevertheless, any such activities must be consistent with the authority's published privacy and other policies so as to avoid scrutiny from bodies such as the US Federal Trade Commission (FTC) for unfair or misleading marketing. (FTC actions potentially would be brought under 15 USCS § 45 (2000) § 45(a), 15 USCS § 52 (2000). For an overview of the FTC's investigative and law enforcement powers see http://www.ftc.gov/ogc/brfovrvw.htm. The FTC has been highly active in scrutinizing site compliance with privacy policies. See *FTC v. Rennert*, in which operators of a group of Online pharmacies that promoted themselves touting medical and pharmaceutical facilities they didn't actually have and making privacy and confidentiality assurances they didn't keep, have agreed to settle FTC charges that their promotional claims were false and violated federal laws [17].) Such policies and practices must also be consistent with applicable state and transnational privacy laws. It should also be noted that the FTC already closely regulates marketing based on endorsements and testimonials that could impact the utilization of a trustmark authority rating by an e-health site [18].

## Risk Management

This purpose of this paper is not to deter those apparently prepared to perform as trustmark authorities. Indeed, the contrary is the case - the potential upside of quality rating for medical sites is too great, and the overall risk-reduction that will be accomplished by a comprehensive, professional trustmark authority is simply too important for such a defeat to be tolerated. Rather, the preceding analysis is offered as a first step in managing the risks attendant with the endeavor.

There are many approaches to such risk management that may be appropriate and require further investigation and possible pilot projects. First, the trustmark authority must have its own quality assurance features that apply to both the centralized and decentralized aspects of the endeavor, and bring consistency to the latter. Another possible internal approach is to incorporate a formal dispute resolution process into the trustmark authority's structure.

As follows from the analysis above, trustmark authorities face a serious yet - at least compared to many businesses - a relatively discrete and, in some regards, even controllable window of liability. However, even assuming a positive result in any litigation, the trustmark authority would still incur considerable defense costs. As such, the utilization of an indemnity strategy such as a third-party liability (errors and omissions) insurance policy that includes a robust duty to defend would be necessary. In this regard, attention should be paid to mandating the scope of coverage taken out by any decentralized bodies, while trustmark authorities will also require financial reserves to handle internal costs associated with defense of suits. Various other risk management techniques will require study. These range from disclosure statements and exculpatory clauses incorporated into the "rating report," to limiting delivery of any report to regional "zones" that feature less aggressive liability rules and related strategies designed to deliver advantageous jurisdictional and choice of law decisions.

Almost inevitably, however, effective risk management likely will require some type of statutory immunity for "Good Samaritan" trustmark authority activities. Regional or transnational in nature, such immunities will also have to deal with the potential extraterritorial reach of the disparate legal systems liability laws. This could require the negotiation of reciprocal safe harbor provisions between major trading groups such as the EU and USA or, with less regard to comity, national or regional provisions denying cross-border enforcement of judgments against trustmark authorities. (An example of such "remedial zoning" is to be found in the UK's "Protection of Trading Interests Act 1980" that limits the UK enforceability of damage awards involving multiple damages - a provision clearly aimed at US antitrust and related laws providing for treble damages (e.g., section 4 of the Clayton Act, 15 USCS § 15).)

## Conclusions

It is naïve to believe that the trustmark authority will exist independently, aloof from the world's legal systems. In seeking to "combat illegal and fraudulent health information on the Internet" [1], a trustmark authority will benefit from public law liability visited on those who misuse its ratings. Such an authority also will be forced to delineate and protect the uses of its certifying marks and other intellectual property from wrongful, misleading, or fraudulent display by health-related web sites. Such issues already are familiar to consumer protection infomediaries in the US (e.g., *Council of Better Business Bureaus, Inc. v. Better Business Bureau, Inc.*, 1999 WL 288669 (N.D.N.Y. Mar 30, 1999); *Better Business Bureau, Inc. v. Medical Directors, Inc.*, 681 F.2d 397 (5th Cir. 1982) which granted a preliminary injunction restraining a weight reduction clinic from representing that their program was approved by rating agency).

If a trustmark authority accused of providing poor advice to a consumer (false positive cases) can pass muster under US law, it should fare at least as well under other systems, be they common or civil law based. In contrast, blacklisting actions brought by the site or content owner being rated against the trustmark authority (false negative cases) are least likely to succeed in the United States because of First Amendment protections for certain types of speech. Thus, a trustmark authority may have greater exposure for defamation-like actions before European courts. (E.g., *Berezovsky v Michaels*[2000] 1 WLR 1004 (HL), facilitating grant of English jurisdiction and service of process in "international" defamation cases. Compare with the US position, the relatively narrow defenses permitted under the UK Defamation Act 1996.)

Working from this baseline of exposure, it will be incumbent on trustmark authorities and the legislative bodies that would endorse them to engineer effective risk management strategies so as not to jeopardize the ameliorative effects of such ratings bodies.
